# Supplementary material for: Experience is central and connections matter: A Leximancer analysis of the research priorities of people with lived experience of mental health issues in Australia
Source: PLOS Ment Health. 2024 Jun 4;1(1):e0000010. doi: 10.1371/journal.pmen.0000010 (PMC12798570; doi:10.1371/journal.pmen.0000010)
Supplement: S1 File — (DOCX) [file pmen.0000010.s001.docx]

**S1 File:** **Leximancer Analysis map iterations and decisions for the ALIVE-ANU Lived Experience Priority Survey Dataset**

The following supplementary file provides the details of the Leximancer analysis decisions and iterations as conducted for the final concept and theme map presented within the manuscript. The manuscript findings centre on the final Leximancer map of themes and concepts (Map 5b) as identified by the analysis of the responses from consumers, carers, consumer-carers from the ALIVE-ANU 2022 priority survey. In this survey, people were asked to share three priorities that they viewed as important for mental health research. Each map is introduced with an explanation of the scale bar % used to interrogate map results and textual responses. The results of each map are discussed with the next iterative decision described.

Map 1a and 1b show the first stage analysis of the .csv file representing concepts (at 30%) and themes (at 30%). This map was the first one that was created from this preliminary analysis of the open-ended responses to the question “share three things that mental health research should focus on” with answers provided into open ended text boxes labelled as priority 1, priority 2 and priority 3. The % scale bar used in the Leximancer maps increases and decreases the number of concepts and themes that are shown on any given map—you can scale in and out of the dataset as required to explore the concepts and themes further and orient to the dataset. For example, if you increase the % number of concepts it means a broader range of interrelated concepts (as they apply within themes) are shown. For themes the % scaling differs—setting to 50% means showing half, reducing this % adds in more themes and increasing to 100% means the primary, main theme is shown. The kind of data one has will define whether broad or tight concepts are explored (reflected in the scale % used) and whether more or less themes are shown on the map.

In the case of this dataset people with lived experience across community settings have contributed sometimes short phrases or one-worded responses and a mix of longer sentences. With this kind of textual data, starting with tighter parameters to explore the concepts within themes is a good first step as it means that the maps will enable the meanings of the concepts in the dataset to be shown to the viewer. For the final map adjusting the themes to 50% for about 30%-50% of concepts and then 100% of concepts to be shown will likely be most meaningful. In these first maps (Map 1a and 1b) no adjustments have been made to Leximancer concept seed settings or terms and sentences that are analysed, and no concepts have been merged.


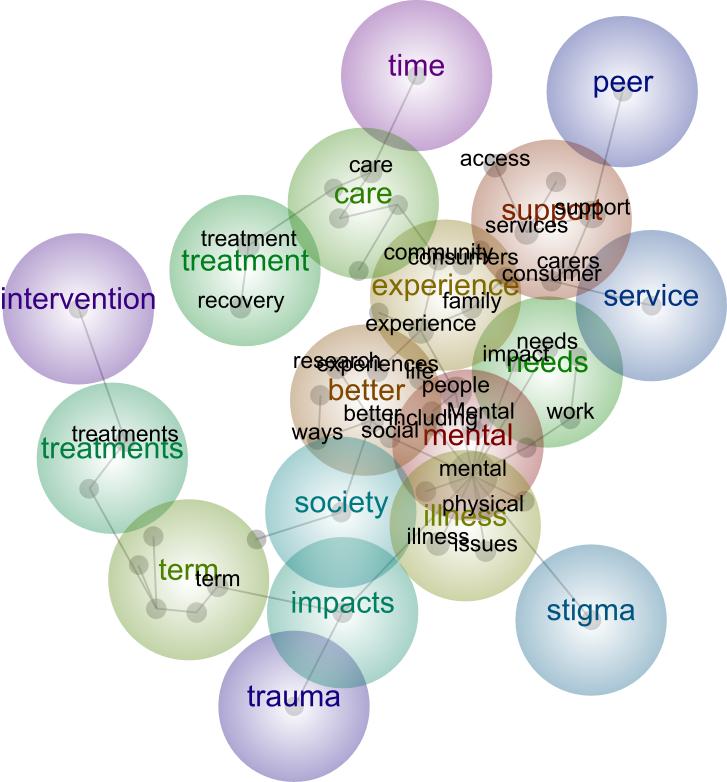

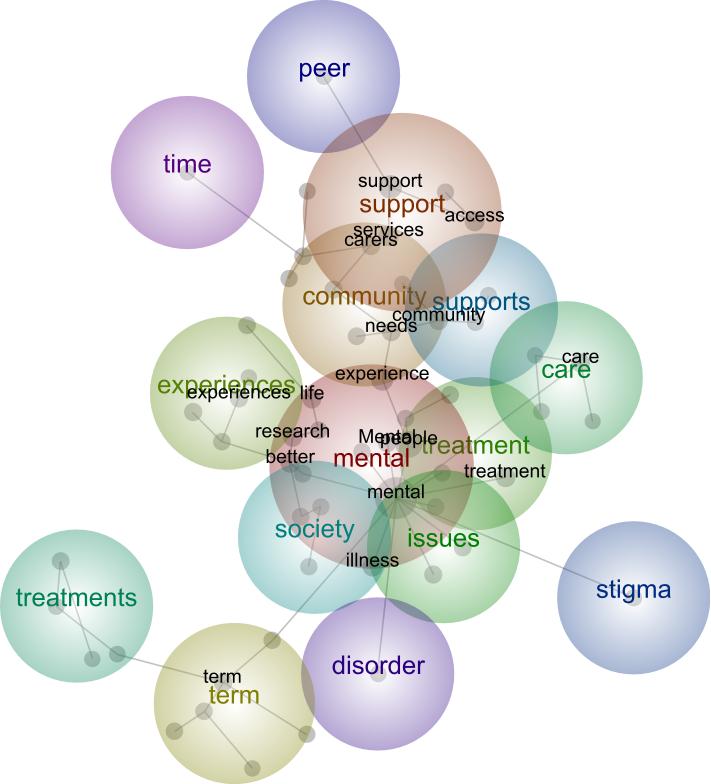


*Map 1a and 1b: Preliminary concept 30% and theme 30% map with no adjustments to data within Leximancer 1a to left looking at the figures and 1b is to the right looking at the figures.*

Map 1a is the first iteration and Map 1b is the second iteration – these were completed twice to check stability of data for the following analyses. Both maps show similarities in themes and concepts indicating the data stability is good. Reviewing the map also shows that many concepts and themes, if left in without adjustment and removal from seed editing lists, would be considered in Leximancer terms as bleached concepts or in other words, ‘overconnected’ terms. By bleached/overconnected concepts we mean that the terms appear so much within text responses that they have become devoid of meaning. Bleached or overconnected concepts are common to general speech patterns and written forms of writing. These include concepts or words such as ‘think’ or ‘kind’. Another term that contributes to bleached or overconnected concepts is stop words which are we put into speech and writing when we are thinking about a response and another sentences is about to start (stop words also create over connection within text responses). When we encounter bleached concepts that are overconnected due to there being stop words in sentences, it is important to remove the stop words too from the concept seed editor list. Removal of bleached concepts and stop words was thus undertaken alongside removal/exclusion of any words that were used within the question asked of respondents—e.g. mental health research and views.

**

Map 2 represents the second iteration of the adjusted map (leaving concepts at 30% of those revealed when looking at 50% of the themes). In this map, the concepts that appeared in the survey question that was asked of respondents were removed and singular and plural words/concepts were merged as were similar concepts. Consumer and carer as concepts were removed in this iteration (for this map and dataset we chose not to use tags to distinguish speakers but the different speaking positions were noted in the textual analysis presented within the paper). This means that the following concepts were removed for the second iteration of Map 2a:

1. The relevant words that were included within the survey question—as it is common that people repeat these words in their text responses—were excluded. The repetition of the word in responses can be seen Map 1a and 1b with “mental” taking the colour red (talked about a lot). In this instance: mental / health / research; and three / things were removed from the concept seed editor list (if these appeared).
2. The words that were likely to be bleached (over connected and devoid of meaning) again, in the question the concepts that also appeared such as, *should, focus and no right or wrong, what’s important and you* - were removed, (if they appeared). In reviewing Map 1a and Map 1b *supports* seems bleached of meaning and interconnected with services so this was removed from the concept seed editor list. In addition to support, *based, need and understanding* and *people* were also removed from the concept seed editor list as these appear to be stop words in sentences used.
3. The following terms were also merged at this stage: experience/experiences; impact/impacts; medication/medications; service/services; treatment/treatments.


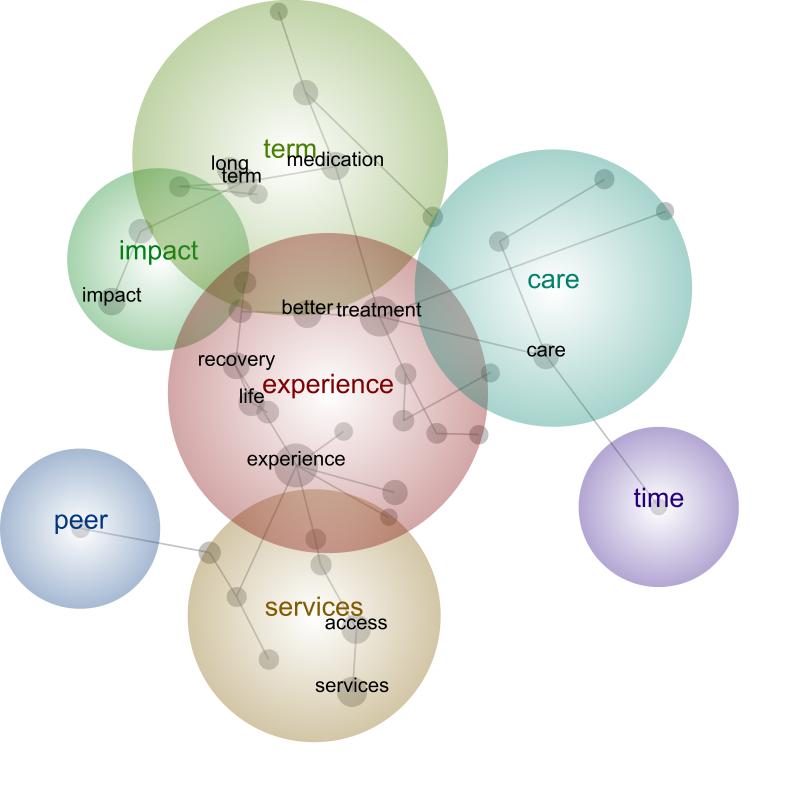


*Map 2a Leximancer iterative adjustment for textual analysis phase concepts (30%) across themes (50%)*

Here in Map 2a, we can see experience is a prominent theme alongside concepts of: *services, term, impact, care, peer* and *time*. It is worth further investigation of the map with all 100% of concepts shown to evaluate whether some additional concepts may or may not need further removal due to over connectedness or being likely stopping words.


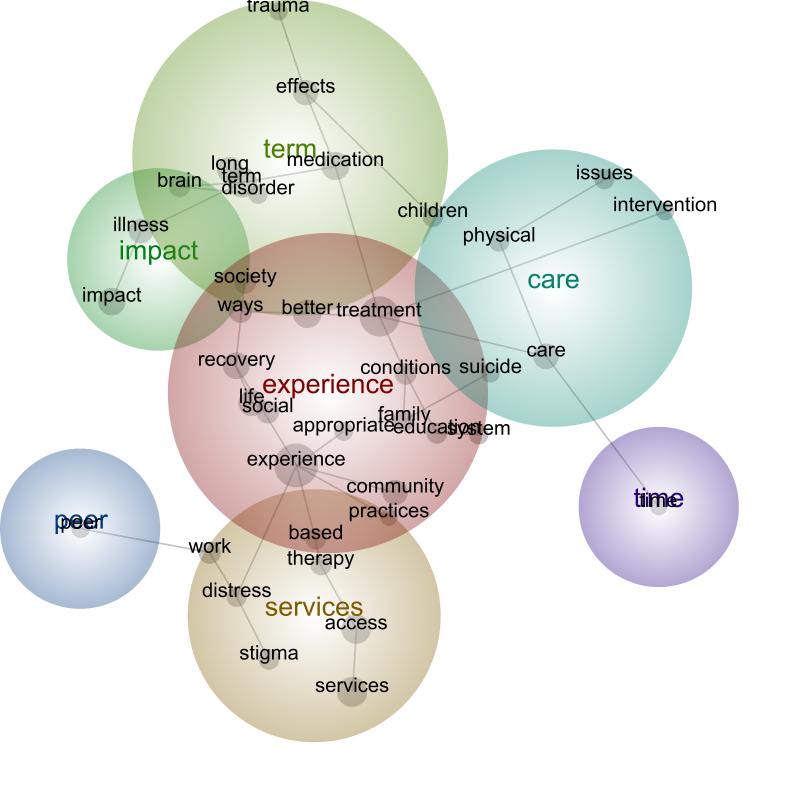


*Map 2b Leximancer iterative adjustment for textual analysis phase concepts (100%) across themes (50%) with merged concepts*

On reviewing Map 2b, it is possible to see that a good deal of concepts could be considered bleached, however, given the nature of the data set removal of too many concepts will remove all meaning – so finding the balance becomes important here. For example, the theme of experience is interconnected and overlapping with all text. This is not surprising since the question asked is about what is important to people and the respondents are people with lived experience. So, this illustrates that people are talking about what is important to them, life experience and recovery and community, for example.

At this juncture in the iterative analysis, the decision was made to leave experience and remove stopping words of *long, term, effects* and *based* within the concepts as they appear on Map 3a and 3b.

**

Map 3a now shows what emerged following removal of further words from the concept seed editor list. The concepts are presented at 30% and themes at 50%.


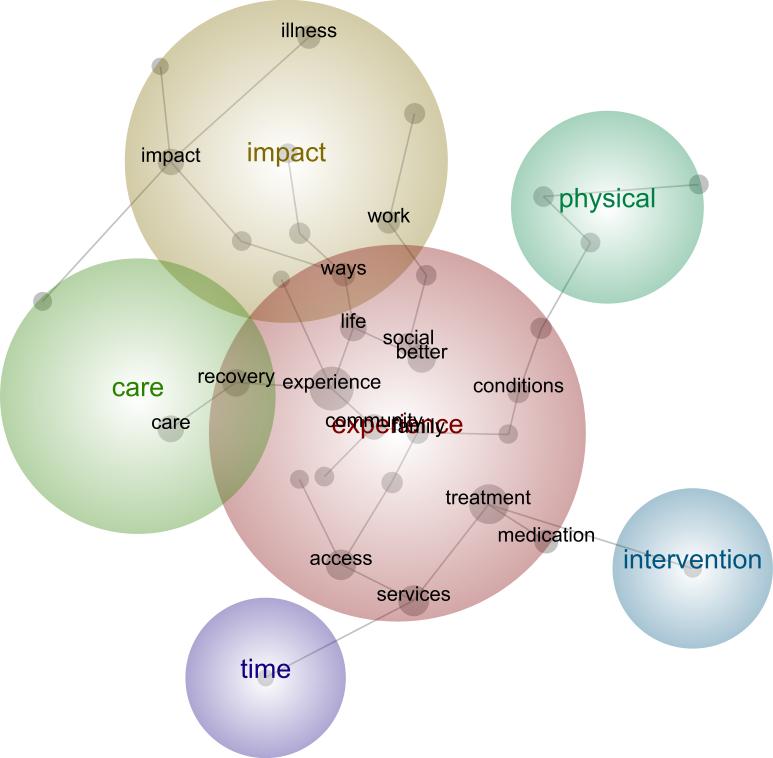


*Map 3a Leximancer iterative adjustment for textual analysis phase concepts (30%) across themes (50%)*

In this map it is possible to see how impact has become a more prominent and interconnected theme with experiences and care has grown in importance. Physical has taken over as impacts has increased in importance and intervention has replaced peer. This is likely to have occurred because responses put peer and intervention together—this is where remembering that some element of textual response examination is always conducted in relation to Leximancer analysis. To explore this further, Map 3b presents the concepts at 100% within 50% for themes.


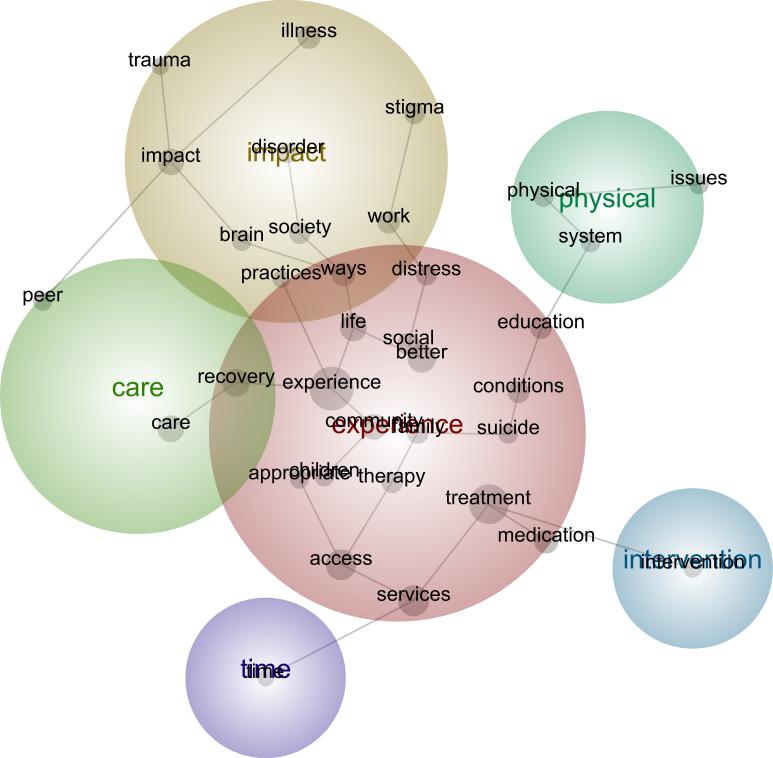


*Map 3b Leximancer iterative adjustment for textual analysis phase concepts (100%) across themes (50%)*

In this map intervention and time appear to be overconnected concepts and the stopping words of issues and ways appear to need to be removed.

**

Map 4a shows 30% of concepts across 50% of the themes.


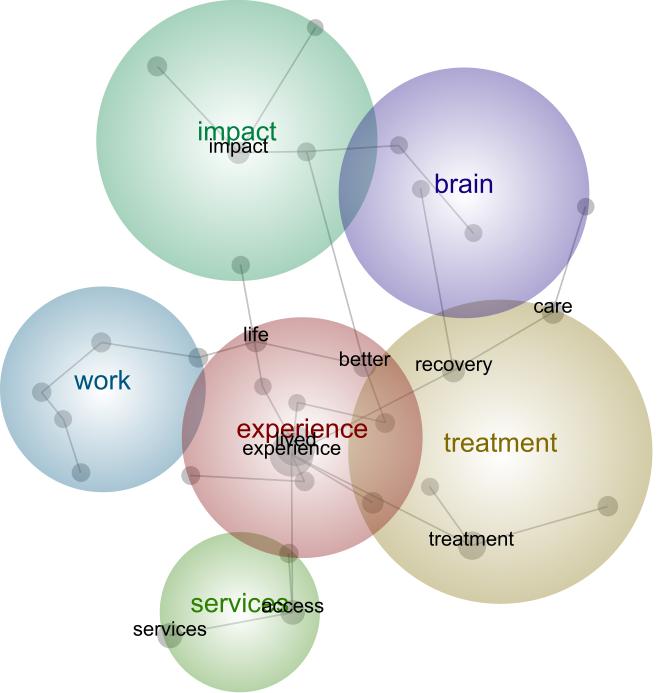


*Map 4a Leximancer iterative adjustment for textual analysis phase concepts (30%) across themes (50%)*

With intervention and time removed as concepts Map 4a shows that work and brain are more prominent withing the textual responses. Further evaluation of the concepts and how they are interrelated is needed to make a final decision on what should be excluded from the concept seed editor further. In Map 4b 100% concepts across 50% of the themes are shown.


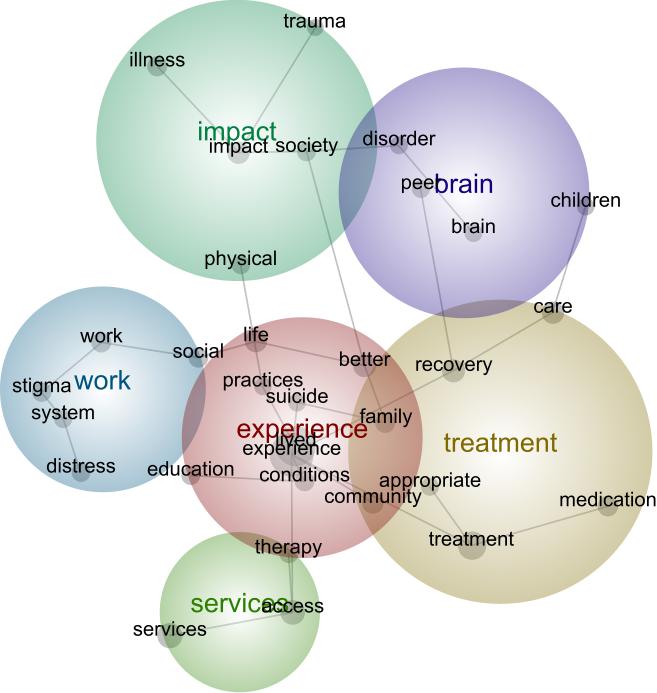


*Map 4b Leximancer iterative adjustment for textual analysis phase concepts (100%) across themes (50%)*

In this map 4b we can see the importance and hence priority area for people who contributed to the survey remains lived and life experience with the concept of experience also connected with better treatment and medication. Following the heat map presentation of results services and access to these are important and the impacts of physical health needs and trauma. Work has now also appeared as a prominent theme in the context of stigma and social contexts.

At this stage Map 4b could be a final theme and concept map used for analysis, however, brain and disorder have now displaced the prominence of peer which was consistent in previous maps and which was deemed important when exploring textual data in relation to the map. The decision was made that brain and disorder were likely to be stopping words or words uses within phrases to describe what it is important so these were excluded from the concept seed editor list. Map 5a presents the outcome.


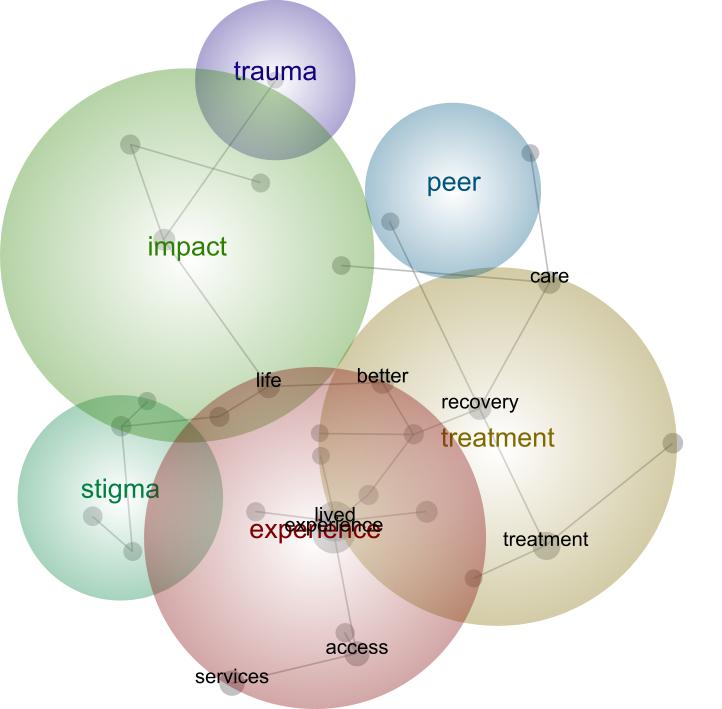


*Map 5a Leximancer iterative adjustment for textual analysis phase concepts (30%) across themes (50%)*

Removal of brain and disorders maintains experience and treatment as the prominent themes (signified by red and brown) and as services has become a concept rather than theme – this is most likely because of the conceptual interconnections of these two areas. Impact is now the third theme of prominence (signified by green), followed by stigma (green blue), peer (blue) and trauma (purple). In Map 5b the final map is shown of 100 % of the concepts that travel across the top 50% of themes.


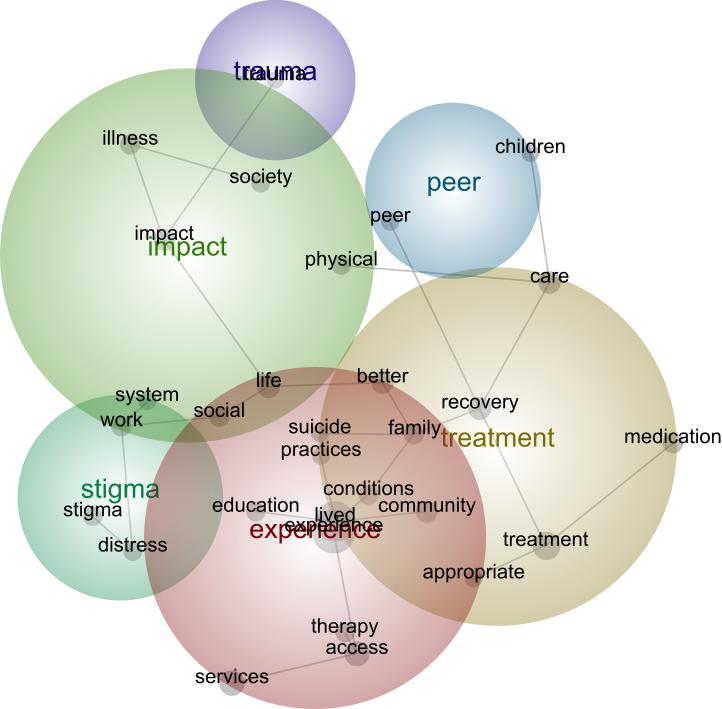


*Map 5b Leximancer iterative adjustment for textual analysis phase concepts (100%) across themes (50%)*

Map 5b represents the final map for analysis of textual data. Since the data used within this analysis ranges from one-word responses to very short and medium length phrases, excluding further concepts from the seed editor would now risk losing the meaning across the full dataset. As this map retains stigma, trauma and impacts as core areas of importance (which is also reflected in the textual data responses) this map was determined to best represent what people in the 2022 survey suggested were the core priority areas of importance for mental health research. These core priority areas are as follows:

Lived Experience – Treatment – Impact – Stigma – Peer(s) - Trauma

The interrelated concepts across these themes are discussed in the findings section of the manuscript.

The following three maps illustrate how different concepts are interconnected when looking at: the primary core theme (e.g. no concepts 0%) which shows experience and illness as the dominant thematic (Map 5c), all themes without 100% concepts (Map 5d) and all concepts without themes (Map 5e).

Map 5c below shows the major theme of experience and illness across all of the 100% interrelated concepts in the textual data.


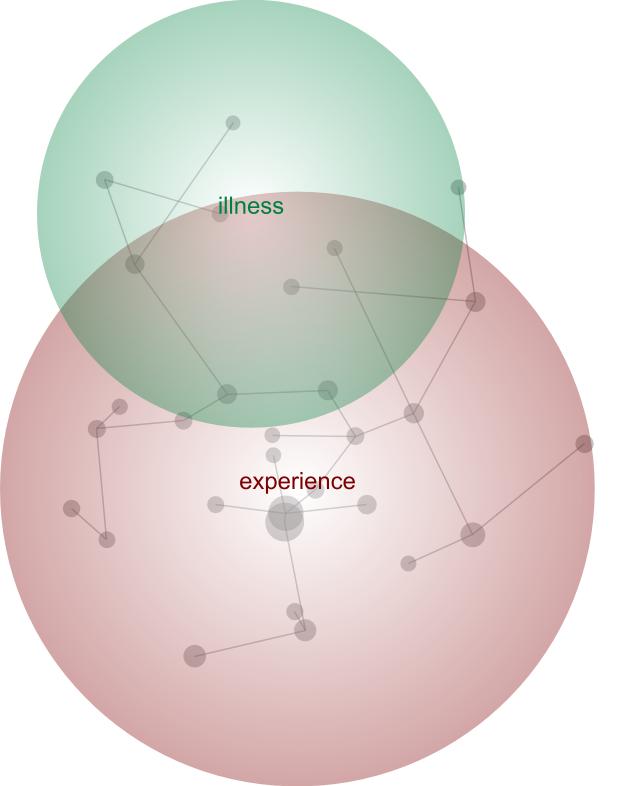


*Map 5c Leximancer iterative adjustment for textual analysis phase concepts (0%) across themes (100%)*

This map shows how experience and illness are core overlapping themes. This representation is reassuring as the responses are from consumers, carers and consumer-carers and therefore illness is a likely concept to appear. Map 5d adds the concepts back into the map at 100% to show how these connected paths tells a textual story.


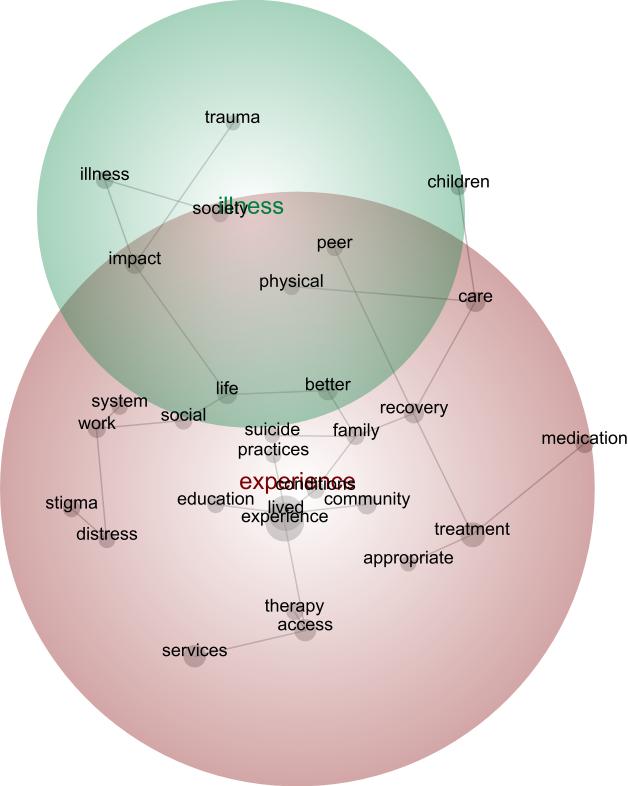


*Map 5d Leximancer iterative adjustment for textual analysis phase concepts (100%) across themes (100%)*


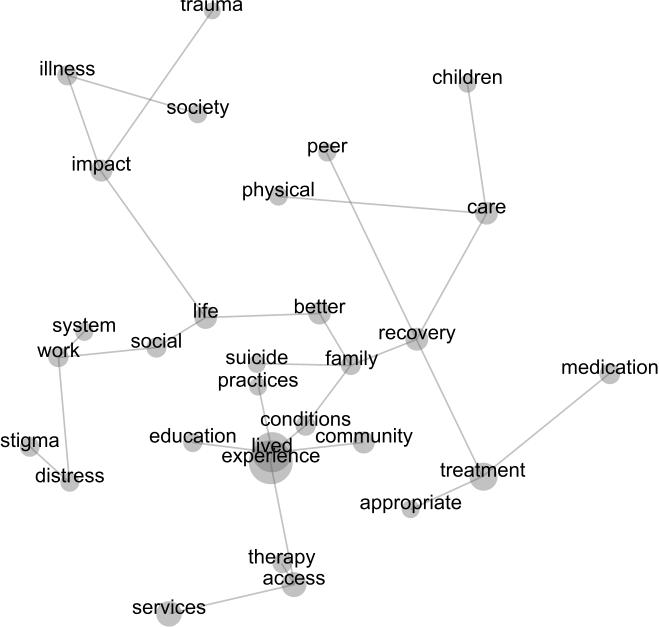


*Map 5e Leximancer iterative adjustment for textual analysis phase concepts (100%) without themes shown.*

Interpretation of final Map 5e can be found in the manuscript of the paper.
