# Supplementary material for: Experience is central and connections matter: A Leximancer analysis of the research priorities of people with lived experience of mental health issues in Australia
Source: PLOS Ment Health. 2024 Jun 4;1(1):e0000010. doi: 10.1371/journal.pmen.0000010 (PMC12798570; doi:10.1371/journal.pmen.0000010)
Supplement: Supplementary file 2 [file pmen.0000010.s002.docx]

ALIVE mental health priority-setting

Start of Block: Core info

Q1 
**Lived Experience (Consumer) and Carer Priorities for Mental Health Research**

 
In this survey you will be asked to list your priorities for mental health research. You will also be asked some questions about yourself. 

 

We are interested in your opinions on research priorities, not what you think other people might think. There are no right or wrong answers.

This survey should take approximately 20 minutes to complete. If you pause and exit the survey, you will be able to return to this website and continue completing your responses for up to one week after you begin. 

 

Your input in this survey will influence the direction of mental health research across The ALIVE National Centre for Mental Health Research Translation and ACACIA: The ACT Consumer and Carer Mental Health Research Unit. 

 

Below is the Participant Information Sheet for this study. You can download and print a copy if you would like to by clicking on the link below. 

[Participant Information Sheet](https://anu.au1.qualtrics.com/CP/File.php?F=F_0uNsKsN5mB5Xxbg)

 

 

 

Funding for this project has been provided by the ACT Government and the National Health and Medical Research Council (APP2002047). 

Q2 I have read and understood the information about this research project. I understand that any information I provide from this point onwards will be included in the research, even if I don’t finish the survey.

- Yes  (1) 

- No  (2) 

Q3 Which of the following roles do you identify? 

 

*A* ***consumer*** *is someone with lived experience of mental ill health, whether or not they have accessed services.* 
*Note: The ALIVE National Centre uses the term 'lived experience' to describe someone with mental ill health, but for this survey we use the term 'consumer'.* 

*A* ***carer*** *is a family member or friend who provides informal (ie. unpaid) support to a mental health consumer.* 

- Consumer  (1) 

- Carer  (2) 

- Consumer and carer  (3) 

- None of the above  (4) 

Display This Question:

If Which of the following roles do you identify?    A consumer is someone with lived experience of m... = Consumer

Or Which of the following roles do you identify?    A consumer is someone with lived experience of m... = Consumer and carer

Q16 How would you describe your mental health needs? (you might like to share diagnoses, supports you access or other names you give to your mental health needs)

- Please enter your description below  (1) __________________________________________________

- I don't have a way to describe my mental health needs  (2) 

Display This Question:

If Which of the following roles do you identify?    A consumer is someone with lived experience of m... = Carer

Or Which of the following roles do you identify?    A consumer is someone with lived experience of m... = Consumer and carer

Q17 How would you describe the mental health needs of the person you support? 

- Please enter your description below  (1) __________________________________________________

- I don't have a way to describe their mental health needs  (2) 

Display This Question:

If How would you describe your mental health needs? (you might like to share diagnoses, supports you... = I don't have a way to describe my mental health needs

Q18 Approximately how long have you experienced distress or mental ill-health for?

________________________________________________________________

Display This Question:

If How would you describe the mental health needs of the person you support?  = I don't have a way to describe their mental health needs

Q19 Approximately how long has the person you are caring for experienced distress or mental ill-health?

________________________________________________________________

End of Block: Core info

Start of Block: Disqualified

Display This Question:

If I have read and understood the information about this research project. I understand that any inf... = No

Or Which of the following roles do you identify?    A consumer is someone with lived experience of m... = None of the above

Or Or What is your age (in years)?  Text Response Is Less Than  18 

Q2 Thank you for your interest in this study. 

 

At this time we are only seeking participation from people *over the age of 18* who identify as *mental health consumers or carers* and who *agree to the terms listed in the participant information sheet*. If you are interested in joining our register or participating in future ACACIA studies, you can find our website [here](https://rsph.anu.edu.au/study/student-projects/acacia-act-consumer-and-carer-mental-health-research-unit). 

For information on the ALIVE National Centre for Mental Health Research please visit: www.alivenetwork.com.au

Skip To: End of Survey If  Thank you for your interest in this study.    At this time we are only seeking participation from... Is Displayed

End of Block: Disqualified

Start of Block: Demographic Information

Q1 What is your age (in years)? 

________________________________________________________________

Q2 What is your gender? 

- Female  (1) 

- Male  (2) 

- I use another term (please describe this term below)  (3) __________________________________________________

- Prefer not to say  (4) 

|  |
| --- |

Q3 What is your postcode? 

________________________________________________________________

Q4 How did you hear about this survey? 

________________________________________________________________

End of Block: Demographic Information

Start of Block: Priorities

Q1 Please share three things that mental health research should focus on - there is no right or wrong answer here just what’s important to you.

- Priority 1  (1) __________________________________________________

- Priority 2  (2) __________________________________________________

- Priority 3  (3) __________________________________________________

Q2 Please share three ways you feel that lived-expertise from either consumer and/or carer (depending on your own experiences) could be included in mental health research?

- Priority 1  (1) __________________________________________________

- Priority 2  (2) __________________________________________________

- Priority 3  (3) __________________________________________________

End of Block: Priorities

Start of Block: Block 2

Q16 **What's next?** 

 

Thank you for contributing your ideas to shape mental health research directions in Australia. We have heard about the importance of these research topics in other work by consumers and carers. We are now keen to know where to put our efforts. 

 

We would love for you to take part in The ALIVE National Mental Health Research Public Prioritisation Exercise! This is a massive public co-design exercise where we will use 'emotion mapping' to combine your responses with others across the nation to co-create a map about how people feel about a list of mental health research areas. This will help us to understand the way people feel about mental health research areas and guide research directions.  

 

Click on the arrow at the bottom of the screen to submit your responses. You can take part in The ALIVE National Mental Health Research Public Prioritisation Exercise by clicking on the link shown in the next page. 

 

 

THANK YOU! 

End of Block: Block 2
