# Supplementary material for: Experience is central and connections matter: A Leximancer analysis of the research priorities of people with lived experience of mental health issues in Australia
Source: PLOS Ment Health. 2024 Jun 4;1(1):e0000010. doi: 10.1371/journal.pmen.0000010 (PMC12798570; doi:10.1371/journal.pmen.0000010)
Supplement: Attachment — Submitted filename: Priority survey 2022 PMEN Response to Reviewers.docx [file pmen.0000010.s003.docx]

Dear Dr Klymchuk

Thank you for the opportunity to submit a revision for manuscript ID PMEN-D-24-00020, titled “Experience is central and connections matter: A Leximancer analysis of the research priorities of people with lived experience of mental health issues in Australia.” Please find below a point-by-point response to the issues raised by the editor and reviewers. Manuscript versions with and without tracked changes have been uploaded as requested, along with new versions of the included figures.

Kind regards

Michelle Banfield

Professor, Lived Experience Research

The Australian National University

**Response to Editor comments**

1. A copy of the dataset has been deposited in the ANU Data Commons, doi: 10.25911/j2dy-p787. This is noted on page 29 of the manuscript and in the submission data.
2. The competing interests statement is located on page 29 of the manuscript. This has been updated to the requested wording.
3. Separate figure files in the requested file format have been uploaded.
4. Reference list has been reviewed and is complete and correct.

**Reviewer #1**

1. It is particularly important that this project takes a lived experience priority position in developing the research priorities, and this is balanced well with the views of carers. I did have a question concerning the design of the overall methodology - was this something that was also co-designed with the relevant community? This is a little unclear.

Response: Clarity has been added on page 8, to reflect that this piece of work fits within the overall program of co-designed work and was also developed by lived experience researchers with input from others with lived experience.

2. I also wondered whether those with lived experience contributed to the research overall - from design through to data collection and onto analysis. Was the community consulted for example in the final themes put forward in the analysis? If not this could be an opportunity for further study, along with understanding whether consumers and carers differed in their priorities.

Response: Thank you for this suggestion. An additional section has been incorporated on page 7, outlining the involvement of people with lived experience across the life of the research. We chose not to add to the complexity of the paper by including group analyses. This has been noted in the limitations and future directions on page 25.

**Reviewer #2**

1. The roles of all the authors to be added in as per recent guidelines on collaborations.

Response: Author contributions are listed on page 29.

2. Same protocol number (perhaps generic) was found (Protocol number 2013/388) in a similar article by the same author - Mental health research priorities in Australia: a consumer and carer agenda

Response: The study was conducted as an amendment to the existing protocol governing the previous published work noted. Australian ethics guidelines discourage duplication of review for studies where the merit and integrity have already been established and there are no additional risks posed by the amendment. A note that this was an amendment has been added on page 7.

3. Broader literature review might help - a good number of the references were self-citations - not bad in itself but limited to finding research in a larger context.

Response: Mental health research priority-setting is a limited field, with few studies focusing on lived experience priorities. We have been conscious to include reference to the major international priority-setting studies alongside our own work. Less than one-third of papers referenced are self-citations, with the remainder representing the majority of priority-setting studies internationally as synthesised in our review (reference 1).
